# Sex Ratio at Birth in India, Its Relation to Birth Order, Sex of Previous Children and Use of Indigenous Medicine

**DOI:** 10.1371/journal.pone.0020097

**Published:** 2011-06-15

**Authors:** Samiksha Manchanda, Bedangshu Saikia, Neeraj Gupta, Sona Chowdhary, Jacob M. Puliyel

**Affiliations:** Department of Neonatology and Pediatrics, St Stephen Hospital, Delhi, India; Aga Khan University, Pakistan

## Abstract

**Objective:**

Sex-ratio at birth in families with previous girls is worse than those with a boy. Our aim was to prospectively study in a large maternal and child unit sex-ratio against previous birth sex and use of traditional medicines for sex selection.

**Main Outcome Measures:**

Sex-ratio among mothers in families with a previous girl and in those with a previous boy, prevalence of indigenous medicine use and sex-ratio in those using medicines for sex selection.

**Results:**

Overall there were 806 girls to 1000 boys. The sex-ratio was 720∶1000 if there was one previous girl and 178∶1000 if there were two previous girls. In second children of families with a previous boy 1017 girls were born per 1000 boys. Sex-ratio in those with one previous girl, who were taking traditional medicines for sex selection, was 928∶1000.

**Conclusion:**

Evidence from the second children clearly shows the sex-ratio is being manipulated by human interventions. More mothers with previous girls tend to use traditional medicines for sex selection, in their subsequent pregnancies. Those taking such medication do not seem to be helped according to expectations. They seem to rely on this method and so are less likely use more definitive methods like sex selective abortions. This is the first such prospective investigation of sex ratio in second children looked at against the sex of previous children. More studies are needed to confirm the findings.

## Introduction

According to the last census, there are only 933 women for every 1000 men in India [1]. Gender bias favoring males is largely responsible for this [2]. Neglect of girls and women resulting in early death [3], [4], [5], female infanticide [6], [7] and more recently, antenatal sex determination and female feticide [8], all contribute to it. Several reports suggest that sex selective abortion became more common in the 1990s [9], [10] after ultrasound machines became available widely in the 1980s [11], [12]. While the Nobel laureate Amratya Sen believes that the pattern of gender inequality shifted from ‘mortality inequality’ to what he calls ‘natality inequality’ due to female feticide after the facility for antenatal sex determination became available [11], [13], others suggest that parents are not substituting pre-natal for post-natal discrimination against girls, but combining the two strategies [14]. The relative contribution of these modes of discrimination, to the unbalanced sex ratio in India, is still unresolved [15]. It is important to resolve this issue so as to target remedies appropriately, before they threaten the stability and security of society [16].

The sex ratio in newborns as registered in the birth register (Registration of Births and Deaths Act 1969), is an indicator of the magnitude of the problem of female feticide as it does not include deaths due to neglect of girl children. However infanticides, in the first few days, are often reported as still-births [15] or not reported at all, within the incomplete birth registration system [7]. Data on sex ratio at birth in hospital records are therefore crucial, to estimate the influence of female feticide on the sex ratio, and which is not affected by other factors like infanticide and neglect of girl children.

A study of hospital birth records over 110 years published by us previously has showed that there was an excess of boys born, if the previous child was a girl. If the first child was girl the sex ratio was 716 girls to 1000 boys and if the first child was boy it was 1140 girls to 1000 boys [17]. This finding apparently runs counter to the normal tendency of biological heterogeneity which results in families having a predilection to have children of the same sex. Biggar et al in Denmark found the probability of having another boy increases to 51.5, 51.6, 52.4 and 54.2 percent for families with previous one, two, three, or four prior boys, respectively [18]. The findings in second children in our previous study points to antenatal interventions and suggest that sex selective abortions are practiced quite commonly to overcompensate for the slight penchant in families to have babies of the same sex. However, we also found this excess of boys in families with a previous girl in a cohort of babies born in 1970s when ultrasound machines were uncommon and the only method of sex determination was amniocentesis and which was not available widely [17]. The methods used by parents to overcome the tendency to have second children of the same sex as the first are not fully understood.

The sex of second children born to parents with a previous girl (or boy) has not previously been studied prospectively in India, to the best of our knowledge. This study was performed to look at the sex ratio in second children depending on the sex of the first child. By studying this prospectively and interviewing mothers it was hoped to gain insights into the practices for sex selection that are in vogue. A priori, it was known that because antenatal sex determination and sex selective abortions are proscribed by law, parents would not admit to these practices, but it was felt they would be more forthcoming about other ‘legal’ methods like use of traditional medicines, to promote birth of babies of the desired sex. The study hypothesis was that families with previous girls are more likely to use traditional medicines for sex selection and that mother taking these medicines are more likely to have a boy child.

## Methods

This prospective study was done from 19 November 2008 to 18 November 2009 in a large hospital known for its maternal and child care services in Delhi. The hospital is century old charitable hospital in the heart of old Delhi catering to the poor and middle class people of the city. All mothers of live born babies delivering in hospital were eligible for inclusion. Informed consent for participation in the study was obtained from the mothers prior to inclusion. The primary focus of interest was mothers delivering their second and third babies. The sex of the child at birth, the sex of previous children was recorded. Mothers were interviewed after they had recovered from the strain of the delivery process – usually 12 hours after delivery. The lady researcher (SM) built up a rapport with the mothers and enquired from her if they knew of any methods or drugs used to get babies of a particular sex. They were also asked if they had utilized any of these methods. The data were recorded on an Excel spreadsheet.

### Sample Size Calculation

The previous data [17] from 2005 in our hospital showed the sex ratio was 629 girls to 1000 boys if the first child was a girl. This gives an odd ratio of 0.49 for second child being female, if the first is female. To detect this odds ratio, in a case control design (case is a girl been born as a second child, control being boy born as a second child if the first is a girl child) with 5% alpha error and 90% power, we needed to study 182 cases and 182 controls. With 80% power this would be 136 cases and 136 controls.

We assumed for simplicity, that the prevalence of exposure – first child being female is 50%. As the remaining 50% first children are presumed to be males, we would need 182+182 cases and 182+182 controls, making a total of 728 babies born as second children, to look at significance in the two sexes separately. About 800 babies are born as second children in our hospital in a year, and therefore we planned to study this over a 1 year period.

### Statistical Methods

Sex ratio was analyzed separately for primigravidas, and in multigravidas according to sex of their previous babies. We looked at the sex of the newborn against the methods they admitted to using for having babies of any particular sex, to see if any method influenced the sex of the baby.

95% confidence intervals (CI) for the sex ratios were calculated. Differences in proportions of the sex ratios were estimated. To look for proportions and their CI, and the difference in proportions with confidence intervals, we used the software ‘Statistics with Confidence’ (www.som.soton.ac.uk).

Approval of the study protocol was obtained separately from the Hospital Research Committee and the Hospital Research Ethic Committee.

## Results

A total of 3795 mothers delivered in the hospital that year. 48 had multiple pregnancies and were not included in the study. 2773 mother who gave birth to singleton babies, participated in the study. In the remaining, data could not be recorded on the account of early discharge from the hospital, before the researcher could interview the mother. A preliminary analysis was done looking at the sex ratio in the group that were not studied and this was no different from the sex ratio in the children studied (difference in proportion 0.009; 95% CI −0.015 to 0.033), suggesting that the drop-out of 974 babies did not bias the study. Further analysis was done on the sample of 2773 mothers who agreed to participate in the study and who signed the consent form.

The results are tabulated in Table 1. The sex ratio in the study sample as a whole was 806 girls to 1000 boys. In primigravida mothers 866 girls were born to every 1000 boys. The sex ratio was 850∶1000 in mothers with one previous child. However, there were only 255 girls to 1000 boys among mothers delivering their third child. This was significantly different from the overall sex ratio (difference in proportion 0.243; 95% CI 0.175 to 0.229).

**Table 1:** Sex Ratio in different Groups.

SerialNumber (Row Number)	Groups[Numbers Girls∶Boys]	Sex Ratio (Number of Girls To 1000 Boys)	Observed Proportion(95% CI)	Difference in proportions between groups (Rows) [95% CI]
**1**	**Study** **Sample**[1238∶1535]	806	0.446(CI 0.428–0.465)	**Row 1:2** 0.018 [CI −0.048 to 0.013]**Row 1:3** 0.013 [CI −0.050 to 0.023]
**2**	**Primiparous Mothers**[754∶870]	866	0.464(CI 0.440–0.489)	**Row 2:3** 0.005 [CI −0.035 to 0.044]**Row 2:6** 0.261 [CI 0.191 to 0.319]*
**3**	**One Previous** **Child**[438∶515]	850	0.460(CI 0.428–0.491)	**Row 3:4** 0.041 [CI −0.013 to 0.094]
**4**	**Previous** **Child:** **Girl**[209∶290]	720	0.419(CI 0.376–0.463)	**Row 4:5** 0.086 [CI −0.148 to −0.022]*
**5**	**Previous** **Child:** **Boy**[229∶225]	1017	0.504(CI 0.459–0.550)	**Row3:5** −0.045 [CI −0.100 to 0.011]
**6**	**Two Previous** **Children**[36∶141]	255	0.203(CI 0.151–0.269)	**Row 1:6** 0.243 [CI 0.175 to 0.229]* **Row 3:6** 0.256 [CI 0.184 to 0.318]
**7**	**Two Previous Girls**[15∶84]	178	0.152(CI 0.094–0.235)	**Row7:9** −0.229 [CI −0.447 to −0.037]*
**8**	**One Girl And Boy** **Previously**[13∶44]	295	0.229(CI 0.138–0.352)	**Row 7:8** −0.077 [CI −0.213 to 0.046]
**9**	**Two Previous** **Boys**[8∶13]	615	0.381(CI 0.209–0.591)	**Row 8:9** −0.153 [CI −0.381 to 0.060]

*Statistically significant difference in proportion.

When looking at the sex ratio in the second babies, taking into account the sex of the first baby, we found that for every 1000 boys there were only 720 girls if the first was a girl and this rose to 1017 girls if the first was a boy. The difference was statistically significant (difference in proportion −0.086; 95% CI −0.148 to −0.022).

There were 184 mothers with two previous children, 106 mother had two previous girls, 21 had two previous boys and 57 had each one girl and one boy. If the two previous children were girls, the sex ratio in the present pregnancy was 178 girls to 1000 boys. Those with two previous boys had a sex ratio of 615 girls for 1000 boys.

Among the 1685 primiparous mothers, only 9 (0.5%) said that they had taken traditional medicine to help them get the baby of a desired sex. However among 978 mothers with 1 previous child, 58 (5.9%) of the mothers had taken these medicines and 54 out of 58 were from 510 mothers with a previous girl (10.6%) and 4 were from 486 mothers with a previous boy (0.8%). Among the 54 with a previous girl child, who had taken medication, there were 26 girls and 28 boys making the sex ratio 928 girls to 1000 boys.

Among 184 mothers with previous two children, 106 had two previous girls and 42 of them had taken medication (39.6%); 21 had 2 previous boys and of them 1 had taken medication (4.8%); 57 had one girl and one boy previously, and of them 3 had taken medication (5.3%).

## Discussion

Some researchers have suggested that the problem of sex selection and the status of women can be expected to be self correcting: as men begin to dramatically outnumber women, women's relative rarity will increase their value; their social status will rise and female offspring will become more desirable [19]. However, studies of societies with high sex ratio and a high proportion of males fail to support this prediction and there is evidence that such societies are disproportionately violent societies [19]. When there is a shortage of women in the marriage market the women can ‘marry up’ inevitably leaving the least desirable men with no marriage prospects [20]. It is a consistent finding across cultures that an overwhelming percentage of violent crime is perpetrated by young unmarried low status males [16]. The need for avoiding this situation is self evident.

We found that the overall sex ratio for deliveries at our hospital was 806 girls to 1000 boys. This is even lower than the sex ratio 865∶1000 we reported from our hospital in the year 2005 [17]. We found that the sex ratio in the second babies, if the first was a girl, was even lower at 720. The sex ratio was 1017 girls to 1000 boys if the first was a boy. The previous retrospective study showed a similar trend where sex ratio was 716 (CI = 672 to 762) if the first child was girl and 1140 (CI = 1072 to 1212) if the first was a boy. The prospectively collected data in this study validated the finding of previous retrospective study and suggest that parents tend to manipulate sex of their offspring. 184 mothers had had two previous children and of these 106 had two previous girls and 21 had two previous two boys. The remaining 57 had had one boy and one girl. Sex ratio for newborns in families with two previous girls was as low as 178 girls to 1000 boys and this empathically underlines the inference of human interference. There were only 21 mothers with 2 previous boys who went on to have a third child compared to 106 who had 2 previous girls. The fact that there were more mothers with two previous girls than there were mothers with two previous boys suggests a tendency among mothers with girls to have more children in the hope of having a boy, while mothers with boy children tend to stop having more babies. In the natural course of events where sex ratio is not manipulated by human intervention, if there is a preference for males, the overall sex ratio will favor girls [18]. This is because of biological heterogeneity which results in families tending to have children of same sex. This phenomenon is not evidenced in India which suggests there is more direct manipulation of the sex ratio in India.

Sex ratio in mothers with 2 previous boys was 615 compared to 1017 in those with 1 previous boy. The small sample size of mothers with 2 previous boys can be the reason for an artifactually low sex ratio here.

Our findings are similar to the findings of Jha et al who looked at sex in second children in a household survey. They found the adjusted sex ratio for the second birth when the preceding child was a girl was 759 per 1000 males. By contrast, adjusted sex ratio for second births if the previous child was a boy was 1102∶1000 [21].

One of the objectives of our prospective study was to enquire into the methods parents may be using to get babies of the desired sex. We were aware that parents are unlikely to incriminate themselves by telling the investigator about antenatal sex determination. However the use of other methods have not been proscribed by law, so we felt it was reasonable to enquire about them in this study. A study by Bandyopadhyay and Singh found that up to 46% mothers use sex selection drugs. They tested 7 samples of such medicines and found 3 contained testosterone one contained progesterone and one a natural steroid [22]. Our study found that more parents who have girls tend to take traditional medicine in the next pregnancy. Overall, some 0.5% mothers took such medication and this percentage increased to 10% if the first child was a girl. 40% of mothers with two girls took such medication.

We found mothers with a previous girl child are more likely to take indigenous medication for sex selection, than mothers with previous a previous boy. Mothers with previous girls were also more likely to have a boy in the next pregnancy. On the face of it may appear that these traditional medicines help mothers to have more boys. However the sex ratio of newborns of mothers taking traditional medicines was 928 girls to 1000 boys. This was much higher compared to the overall ratio of 720 girls to 1000 boys in mothers with one previous girl child. It was also higher than the overall sex ratio of 806∶1000. This suggests that the subset of mother who took these medicines perhaps relied on them and it prevented them from resorting to techniques like antenatal sex determination and sex selective abortions.

Our study has one notable weakness. It relates to sex ratio of children born in a hospital. According to the National Family Health Survey 3 (2005–2006), 60% of all deliveries in India take place at home and outside of the medical institutions. Our data cannot therefore be said to be representative of India. It may be argued that if the sex of the child is known antenatally, there is a greater chance that male fetuses will be brought to the hospital for delivery and this could alter the ratio. This may be seen as an antenatal extension of the practice wherein boys are presented earlier in their illness and more frequently to the hospital [4]. However the data from second children delivered at this hospital shows that the majority of children are girls if the previous child was a boy, and this militates against the suggestion that boy fetuses are selectively bought to hospital for delivery.

In summary our study suggests that in spite of Pre-natal Diagnostic Techniques (Regulation and Prevention of Misuse) Act 1994, the sex ratio at birth continues to fall. Evidence from the second children clearly suggests this is the result of human interventions. The use of traditional medicines in mother with previous girl children does not influence the sex ratio and mother who use traditional medicine perhaps do not employ other means to manipulate the sex ratio. The exact method used by some parents to influence the sex of their children is yet not clear. The impulse is to blame it all on ultrasound machines but our previous work on sex ratio in second babies has shown that the skewed sex ratio in second children was common even in the 1970s before ultrasound machines were freely available. More research is needed to elucidate this.
